# More Tinkering

**Published:** 1857-06

**Authors:** 


*More Tinkering.*—At the last session of the American Medical Association, at Nashville, pending a discussion of the subject of medical education, Dr. Curry offered the following resolutions:

*Whereas*, The subject of Medical Education has been committed at each annual session to standing committees, and various suggestions have been proposed which the Association has adopted, and recommended to private instructors and to the medical colleges,

*Resolved*, That a committee of five be appointed by the committee on nominations, as a special committee, to be composed of members who are in no respect connected with any medical school, to devise *a System of Medi-*

*cal Instruction*, to be presented for the consideration of this Association at its annual session in 1858.

*Resolved*, That the proposed system shall set forth a uniform basis upon which our medical institutions shall be organized as well as have reference to the best mode of securing the preparatory medical instruction to the student, and that consequently the legitimate subjects to be embraced in said system will include Primary Medical Sch ols, the number of Professorships in Medical Colleges, the length and number of terms during the year, the requisite qualifications for graduation, and such other subjects of a general character as to give uniformity to our medical system, and preserve harmony and friendly intercourse in the ranks of the profession.

*Resolved*, That, upon the adoption of the proposed system by the association, all institutions which may conform to it shall be entitled to representation at the annual session of this association, and none others.

These resolutions passed, and the following committee were named to carry them out:

James R. Wood, of New York; George R. Grant, Memphis, Tenn.; John Watson, New York; C. B. Nottingham, Macon, Ga.; Rene La Roche, Philadelphia.

What all this may result in is past our power of prophecy. We would like to see stringent rules enacted, requiring a high standard for the degree; and requiring, also, on the part of the schools, certain requisite advantages in the way of clinical opportunity, number of faculty and length of term. We would willingly stand or fall by such a system, but so long as the great schools of New York and Philadelphia hang back, let no man attempt to dragoon the provincial schools into a movement in which they are expected to ruin themselves for the benefit, not of the profession, but of these same metropolitan schools.

We honor those schools, and have no enmities with them, but let it be understood that in these much talked of reformatory movements they must take the lead. They have wealth and all the advantages which are attractive to students; they can afford to lead off. We of the provinces will follow in this matter, we choose to be secondary, inasmuch as what little experience we have had in a similar movement only resulted in benefit to them and injury to ourselves. But we know that the provincial schools will not flinch, and the Philadelphia and New York centres once taking a firm step for reform, will find us along side them. Let us see.

